# Analyzing the serum biochemical factors that influence early neurological deterioration in ischemic Stroke patients and developing a nomogram prediction model

**DOI:** 10.5937/jomb0-51371

**Published:** 2025-01-24

**Authors:** Zhan Xiaoni, Xu Yunyun, Ma Rongrong

**Affiliations:** 1 Wenzhou Medical University, Dongyang Peoples Hospital, Department of Neurology, Zhejiang, China

**Keywords:** serum biochemical factors, early neurological deterioration, nomogram prediction model, biohemijski faktori u serumu, rano neurološko pogoršanje, normogram model za predikciju

## Abstract

**Background:**

To investigate the risk factors associated with early neurological deterioration (END) in ischemic stroke (IS) patients and develop a predictive nomogram model.

**Methods:**

General clinical data from 220 IS patients treated between December 2022 and November 2023 were collected for observation. The study's inclusion and exclusion criteria select patients aged 18+ with a first-time diagnosis of IS who undergo lab tests within 24 hours of admission while excluding those with multiple organ dysfunction, sensory impairments, coagulation disorders, or other serious medical conditions. Based on the National Institutes of Health Stroke Scale (NIHSS) in the United States, patients were categorized into two groups: END (n=69) and non-END (n=151). Both groups' basic demographics, medical history, and biochemical test results were compared. Influencing factors were identified using the least absolute shrinkage and selection operator (LASSO) method, and these variables were included in a multivariate logistic regression analysis to construct a nomogram for predicting END in IS patients. Model performance was evaluated using internal validation with the Bootstrap method, assessing discrimination, calibration, and clinical validity.

**Results:**

Factors such as history of diabetes, fasting plasma glucose (FBG), triglyceride (TG), homocysteine (Hcy), and C-reactive protein (CRP) were identified as single factors for early functional deterioration in IS patients (P<0.05). A logistic regression model was established with END as the dependent variable and significant single factors (P<0.05) as independent variables. The results indicated that diabetes history (OR=1.398, P=0.301), TG (OR= 6.149, P<0.05), ASPECT score (OR=7.641, P<0.05), FBG (OR=2.172, P<0.05), CRP (OR=1.706, P<0.05), NIHSS score 7 days post-admission (OR=1.336, P<0.05), and Hcy (OR=1.425, P<0.05) were independent risk factors for END in IS patients (P<0.05). ROC analysis showed an ASPECT area under the curve of 0.910 (95% CI:0.864 to 0.944), with 84.06% sensitivity and 86.09% specificity. Hcy had an area under the curve of 0.808 (95% CI:0.750 to 0.858), with 79.71% sensitivity and 70.20% specificity. FBG had an area under the curve of 0.847 (95% CI:0.793 to 0.892), with 69.57% sensitivity and 95.36% specificity. TG had an area under the curve of 0.937 (95% CI: 0.896-0.965), with 91.30% sensitivity and 82.78% specificity. NIHSS had an area under the curve of 0.857 (95% CI: 0.803-0.900), with 89.86% sensitivity and 70.20% specificity. A nomogram model for END risk prediction was constructed based on the logistic regression analysis results, assigning preliminary scores for each of the 9 predictive factors. The total score, ranging from 0-100 points, was used to predict END risk in patients (0-100%). The constructed nomogram model showed that ASPECT was 59.2, Hcy was 84.0, FBG was 61.4, TG7.0 mmol/L was 39.4, and NIHSS was 98.1 with a total score of 345.7 which predicted the risk of END at 68.9%.

**Conclusions:**

ASPECT, Hcy, FBG, TG, and NIHSS are independent factors influencing END after IS. On this basis, a visual predictive nomogram model is constructed to predict the risk of END in patients accurately.

## Abstract (sr)

**Uvod:**

Cilj je bio ispitivanje faktora rizika povezanih sa ranim neurološkim pogoršanjem (END) kod pacijenata sa ishemijskim moždanim udarom (IS) i razvoj predikcionog modela nomograma.

**Metode:**

Za potrebe posmatranja prikupljeni su opšti klinički podaci od 220 pacijenata sa ishemijskim moždanim udarom (IS) lečenih u periodu od decembra 2022. do novembra 2023. godine. Kriterijumi za uključivanje i isključivanje iz studije obuhvatili su pacijente starije od 18 godina sa prvom dijagnozom IS-a, koji su podvrgnuti labo ratorijskim testovima u roku od 24 sata od prijema, dok su isključeni pacijenti sa višestrukom disfunkcijom organa, senzornim oštečenjima, poremečajima koagulacije ili drugim ozbiljnim medicinskim stanjima. Na osnovu Nacionalne skale za moždani udar (NIHSS) u Sjedinjenim Državama, pacijenti su podeljeni u dve grupe: END (n = 69) i non-END (n = 151). Upoređeni su osnovni demografski podaci, medicinska istorija i rezultati biohemijskih testova obe grupe. Faktori koji utiču identifikovani su pomoču metode najmanje apsolutne kontrakcije i selekcije operatora (LASSO), a ove varijable su uključene u multivarijatnu logističku regresionu analizu radi konstrukcije nomograma za predviđanje END kod pacijenata sa IS-om. Performanse modela su procenjene internom validacijom pomoču "Bootstrap" metode, uz ocenu diskriminacije, kalibracije i kliničke validnosti.

**Rezultati:**

Faktori kao što su istorija dijabetesa, nivo glukoze u krvi na prazan stomak (FBG), trigliceridi (TG), homocistein (Hcy) i C-reaktivni protein (CRP) su identifikovani kao pojedinačni faktori za rano funkcionalno pogoršanje kod pacijenata sa ishemijskim moždanim udarom (P<0,05). Logistički regresioni model je uspostavljen sa END kao zavisnom varijablom, a značajni pojedinačni faktori (P<0,05) kao nezavisne varijable. Rezultati su pokazali da su istorija dijabetesa (OR=1,398, P=0,301), TG (OR=6,149, P<0,05), ASPECT skor (OR=7,641, P<0,05), FBG (OR=2,172, P<0,05), CRP (OR=1,706, P<0,05), NIHSS skor 7 dana nakon prijema (OR=1,336, P<0,05) i Hcy (OR=1,425, P<0,05) nezavisni faktori rizika za END kod pacijenata sa ISom (P<0,05). ROC analiza je pokazala da ASPECT ima površinu ispod krive od 0,910 (95% CI: 0,864 do 0,944), sa osetljivošču od 84,06% i specifičnošču od 86,09%. Hcy je imao površinu ispod krive od 0,808 (95% CI: 0,750 do 0,858), sa osetljivošču od 79,71% i specifičnošču od 70,20%. FBG je imao površinu ispod krive od 0,847 (95% CI: 0,793 do 0,892), sa osetljivošču od 69,57% i specifičnošču od 95,36%. TG je imao površinu ispod krive od 0,937 (95% CI: 0,896-0,965), sa osetljivošču od 91,30% i specifičnošču od 82,78%. NIHSS je imao površinu ispod krive od 0,857 (95% CI: 0,803-0,900), sa osetljivošču od 89,86% i specifičnošču od 70,20%. Nomogram model za predikciju rizika od END-a je konstruisan na osnovu rezultata logističke regresione analize, dodeljujuči preliminarne ocene za svaki od 9 prediktivnih faktora. Ukupni rezultat, u rasponu od 0 do 100 poena, koriščen je za predviđanje rizika od END-a kod pacijenata (0-100%). Konstruisani nomogram model je pokazao da je ASPECT bio 59,2, Hcy 84,0, FBG 61,4, TG>7,0 mmol/L bio 39,4, a NIHSS 98,1, sa ukupnim rezultatom od 345,7, što je predviđalo rizik od END-a od 68,9%.

**Zaključak:**

ASPECT, Hcy, FBG, TG i NIHSS su nezavisni faktori koji utiču na END nakon IS-a. Na osnovu toga je konstruisan vizuelni prediktivni nomogram model za tačno predviđanje rizika od END-a kod pacijenata.

## Introduction

Ischemic stroke (IS) is characterized by necrosis of brain tissue due to the narrowing or blockage ofblood-supplying arteries, such as the carotid and vertebral arteries, resulting in an inadequate cerebral blood supply [1]. The pathogenesis of the disease is closely related to cerebrovascular lesions, such as long-term hypertension, hyperlipidemia, diabetes, and vascular ageing. Lesions may induce the formation of cerebral aneurysms, which undoubtedly greatly increases the risk of cerebral haemorrhage [2]. Factors such as obstruction of blood flow, vascular abnormalities, and primary haemorrhage may also cause IS [3]. The clinical manifestations of IS vary, including headache, nausea, vomiting, dyskinesia, sensory disturbance, speech disorder, and disturbance in consciousness [4]. Specifically, headache is the most common, mainly caused by increased intracranial pressure and brain compression from blood clots [5]. Nausea and vomiting are also typical symptoms of IS, especially in the early stage of haemorrhage. Dyskinesia, sensory disturbance, and speech disorder may be the result of compression and damage to cranial nerves caused by haemorrhage [4] [6].

Following a stroke, patients typically experience a dynamic change in their neurological status [7].Early neurological deterioration (END) describes the decline in neurological function occurring within hours or days after the onset of acute ischemic stroke [8]. This condition is also called ‘progressive stroke’, ‘early progression of stroke’, ‘progressing stroke’, and ‘evolutionary stroke’. According to a recent study, approximately one-seventh of hospitalized patients with IS may experience END within 24 hours after onset [9]. The incidence of END peaked within 24 hours after admission and then gradually declined [10]. Exacerbation is manifested as aggravation and expansion of neurological deficits, which are important for patients’ rehabilitation process and prognosis. This deterioration primarily results from reduced blood supply to the brain due to cerebrovascular stenosis or obstruction, which leads to hypoxia and abnormal brain cell metabolism, eventually causing cell death [11]. At stroke onset, nerve cells in the affected area begin to die, significantly impairing neurological function. Post-stroke, thrombus formation at the site of the initial vascular narrowing or blockage can further exacerbate vascular obstruction, increasing ischemia and hypoxia in brain tissue and thereby triggering further neurological decline [12].

Research has demonstrated that addressing risk factors for early END can markedly reduce its incidence. However, current prediction methods mainly rely on single risk factors and lack comprehensive scientific accuracy. Therefore, to predict the characteristics of END onset, researchers should consider multiple risk factors together to develop a more targeted and effective predictive tool [13]. The field increasingly focuses on END prediction models, with a trend toward improving accuracy through the joint analysis of various risk factors. Researchers are concentratingon common risk factors like age, gender, hypertension, diabetes mellitus, and hyperlipidemia to develop more reliable prediction models [14].

The nomogram presents the predicted probabilities simply and intuitively, and clinicians can quickly assess patients’ risk of developing END through the END nomogram [15]. The nomogram can comprehensively consider multiple factors of patients, including age, gender, disease history, and laboratory test results, to provide personalized END risk assessment for each patient. Through the END nomogram, physicians can identify high-risk patients and develop targeted treatment regimens, thus reducing the incidence of END and improving patient prognosis. The END nomogram can also be used in clinical studies to compare the effects of different treatment regimens and promote the scientificity and reliability of clinical studies. This paper constructs an individualized nomogram prediction model by screening the independent risk factors related to END to assist clinical assessment and formulate positive and effective intervention strategies.

## Materials and methods

### Research subjects

The basic clinical data of 220 patients with IS admitted from December 2020 to November 2023 were collected by retrospective analysis and used as the subjects for observation in this study. Patients or their families will provide written informed consent. Inclusion criteria: ① Patients meeting the diagnostic stan dards for IS; ② Those aged > 18 years old; ③ Those with the first onset of the disease; ④ Those who complete various laboratory tests within 24 hours after admission. Exclusion criteria: ① Combined with multiple organ dysfunction such as heart and kidney; ② Combined with hearing, visual acuity, or consciousness impairment; ③ Transient cerebral ische - mia and intracranial haemorrhage episodes; ④ Combined with coagulation disorders; ⑤ Combined with a malignant tumour, tuberculosis, and other diseases; ⑥ Combined with a systemic immune reaction.

### Survey tools

### General data

Gender, age, time from onset to admission, etc.

### END diagnostic criteria

According to the National Institutes of Health Stroke Scale (NIHSS) assessment (Schnabel et al., 2021) in the United States, an increase of ≥2 points in the NIHSS score compared with the baseline within 48 hours of admission indicates END.

### Past medical history

①Hypertension: (Monitored systolic blood pressure ≥140 mmHg or diastolic blood pressure ≥90mmHg in a non-resting state on the same day), ② Diabetes mellitus (2-hour postprandial blood glucose ≥11.1 mmol/L or fasting blood glucose ≥7.0 mmol/L), ③ Hyperlipidemia (triglycerides (TG) ≥7.0 mmol/L or high-density lipoprotein cholesterol (HDLC) <1.04 mg/dL). ④ Smoking history (cumulative or continuous smoking for ≥6 months and at least 1 cigarette per day), ⑤ Drinking history (consecutive 6 months and weekly alcohol consumption ≥50 mL).

### Laboratory indicators

① Fasting plasma glucose (FBG): (4.4–6.1 mmol/L); ② triglyceride (triglyceride, TG): (<5.18 mmol/L); ③ Low-density lipoprotein cholesterol (LDL-C) ( 3.37 mmol/L); ④ High-density lipoprotein cholesterol (HDL-C): (Normal values range from 1.16 mmol/L to 1.42 mmol/L for adult males and 1.29 mmol/L to 1.55 mmol/L for adult females); ⑤ Homocysteine (Hcy): (5.08–15.39 mmol/L); ⑥ Creactive protein (CRP): 0 mg/L–10 mg/L; ⑦ Hemoglobin (Hb): (120–160 g/L for adult males and 110–160 g/L for adult females); ⑧ Red blood cell count (RBC): (Normal range is 4–5.5×1012/L inmales and 3.5–5.0×1012/L in females); ⑨ Platelet count (PLT): ((100–300) × 109/L); ⑩ Neutrophil ratio (NE): 50%–70%; ⑾ Lymphocyte ratio (LY): 20%–40%.

### NIHSS

The NIHSS includes 13 aspects, such as visual field, level of consciousness, ataxia, gaze, upper and lower limb movements, sensory functions, language, and facial paralysis, with a total possible score of 0–42. Higher scores indicate more severe neuroengineering function impairment.

### Severity of disease

The Alberta Stroke Program Early CT Score (ASPECTS) is a CT scoring method designed to evaluate early ischemic changes in ischemic stroke patients’ middle cerebral artery territory. This system rapidly and reliably assesses ischemic lesions and aids in predicting the effectiveness of thrombolytic therapy and the long-term prognosis of patients. ASPECTS divides the cerebral cortex into several regions, assigning scores to these areas. These typically include four subcortical regions (caudate nucleus C, lentiform nucleus L, internal capsule IC, and insula I) and six cortical regions (M1 to M6). Each region receives one point, with a total possible score of ten. During scoring, one point is subtracted for each region exhibiting lesions. Thus, a normal brain CT score is 10, while a score of 0 indicates extensive infarction in the middle cerebral artery territory [16].

### Construction and validation of the nomogram prediction model

Univariate and multivariate logistic regression analyses were conducted on the general patient data collected, and independent predictive risk factors identified from the multivariate analysis were used to build a nomogram prediction model. The model’s calibration was highly evaluated using the Hosmer-Lemeshow goodness-of-fit test. The Consistency Index (C-index) further validated the model’s consistency, indicating high stability in the prediction process. The model’s discrimination was fully confirmed through receiver operating characteristic curve (ROC) evaluation. To ensure reliability and stability, internal validation was performed using the Bootstrap method, with the model undergoing a series of tests by re-sampling 800 times. Simultaneously, an external validation set was used to evaluate the model’s performance in real-world applications.

### Statistical methods

The clinical data of IS patients were analyzed using SPSS 25.0 and R (version 3.6.2). Normally distributed measurement data were presented as mean ± standard deviation, skewed distribution data were described by median (P25, P75), and enumeration data were expressed by number of cases, percentage, and rate. During data analysis, the least absolute shrinkage and selection operator (LASSO) regression was employed to screen for potential factors contributing to early neurological deterioration in IS patients. This method identifies important variables, providing a solid basis for subsequent clinical intervention and prevention.

To evaluate the performance of the LASSO regression model, we used the area under the receiver operating characteristic (ROC) curve (AUC) to assess its discriminability. The AUC value ranges from 0 to 1, with values closer to 1 indicating better model performance. We also used the Hosmer-Lemeshow goodness of fit test to evaluate the model’s calibration.

The variables identified by LASSO regression were then included in a multivariate logistic regression analysis to explore their relationship with early neurological deterioration in IS patients further. The logistic regression model was used to estimate the odds ratios (ORs) and 95% confidence intervals (CIs) for each independent variable. The OR represents the change in the odds of the outcome variable (early neurological deterioration) for a one-unit change in the independent variable while holding all other independent variables constant. The 95% CI provides a range of plausible values for the true OR.

To evaluate the performance of the logistic regression model, we used the ROC curve analysis to assess its discriminability. The AUC value was calculated to evaluate the model’s ability to distinguish between patients with and without early neurological deterioration. We also used the C-index to evaluate the model’s generalization ability.

A nomogram prediction model was constructed by quantifying the variables and building models. The nomogram model was used to predict the risk of IS post-END based on the total scores. The performance of the nomogram model was evaluated using the Hosmer-Lemeshow goodness of fit test, ROC curve analysis, and C-index.

In evaluating model performance, internal validation based on the Bootstrap method was used tocomprehensively assess the model’s discriminability, calibration, and clinical validity using the Consistency Index (C-index), calibration curve, and Decision Curve Analysis (DCA). Discrimination, a key measure of the model’s predictive ability, was evaluated by comparing actual observed values with predicted values, recording the regression coefficient (β), odds ratio (OR), and its 95% confidence interval and Pvalue for each included variable. A P-value <0.05 was used as the criterion for statistical significance, and all tests were two-sided.

Calibration assesses the consistency between predicted and actual results, with calibration curves intuitively showing the relationship between predicted and actual risks. The closer the calibration curve is to the ideal state, the better the model’s calibration. Clinical validity refers to the model’s value in practical clinical application, with DCA helping to understand the model’s predictive ability in terms of patient survival benefit at different thresholds.

To ensure the accuracy of data analysis, it was assumed that the data were missing at random, and missing values were processed using a “mice” package. Multiple interpolation through chain equations allows for a more accurate estimation of model parameters, thus improving the reliability of model evaluation.

## Results

The study population comprised 220 IS patients, with a male-to-female ratio of 132:88 (60.00% vs 40.00%). The majority of patients (137, 62.27%) were older than 65 years, while 83 patients (37.73%) were 65 years or younger. Early neurological deterioration (END) was observed in 69 patients (31.36%), while 151 patients (68.64%) did not experience END. Based on the chi-square test for qualitative variables and independent t-test for continuous ones, significant differences were observed between the END and non-END groups in several variables. The END group had a higher prevalence of diabetes (69.57% vs 24.50%, χ^2^=40.562, p<0.01) and higher levels of fasting blood glucose (FBG) (8.59±3.13 mmol/L vs 4.99±1.36 mmol/L, t=11.908, p<0.01), triglycerides (TG) ≥7.0 mmol/L (60.86% vs 25.17%, χ^2^=26.089, p<0.01), homocysteine (Hcy) (13.59± 3.43 μmol/L vs 9.37±3.41 μmol/L, t=8.501, p<0.01), and C-reactive protein (CRP) (8.25±2.97 mg/L vs 4.26±2.39 mg/L, t=10.623, p<0.01). Additionally, the END group had higher NIHSS scores on admission (8.46±2.82 vs 3.62±2.63, t=9.224, p<0.01) and lower ASPECT scores (6.50±0.85 vs 8.64±0.81, t=17.901, p<0.01). Other comparisons were not statistically significant. See Table 1 for details. So, For statistical analysis, the dependent variable END (early neurological deterioration) was assigned a binary value of 0 (No) or 1 (Yes). The independent variables were also assigned as follows: diabetes was coded as 0 (No) or 1 (Yes); triglycerides (TG) were categorized as 0 (<7.0 mmol/L) or 1 (≥7.0 mmol/L); and the original data values were used for ASPECT score, fasting blood glucose (FBG), C-reactive protein (CRP), NIHSS score, and homocysteine (Hcy).

**Table 1:** Univariate analysis of early neurological deterioration in patients with IS. Notes: END stands for Early Neurological Deterioration; FBG is fasting blood glucose; TG represents triglycerides; LDL-C refers to Low-density lipoprotein cholesterol; HDL-C indicates High-density lipoprotein cholesterol; Hcy is homocysteine; CRP denotes C-reactive protein; Hb stands for Hemoglobin; RBC is Red blood cell count; PLT represents Platelet count; NE refers to Neutrophil ratio; LY is the Lymphocyte ratio; NIHSS is the National Institutes of Health Stroke Scale in the United States; ASPECT stands for CT score.

Item	END group<br>(*n*=69)	Non-END group<br>(n=151)	t/χ^2^ value	P value
Hypertension, n (%)				
Yes	53(76.81)	107(70.86)	0.846	0.358
No	16(23.19)	44(29.14)		
Diabetes, n (%)				
YES	48(69.57)	37(24.50)	40.562	<0.01
No	21(30.43)	114(75.50)		
Hyperlipidemia, n (%)				
Yes	3(4.35)	7(4.64)	0.009	0.924
No	66(95.65)	144(95.36)		
Smoking history, n (%)				
Yes	21(30.43)	53(35.10)	0.462	0.497
No	48(69.57)	98(64.90)		
Drinking history, n (%)				
Yes	18(26.09)	44(29.14)	0.218	0.641
No	51(73.91)	107(70.86)		
Time from Onset to Admission (x̄±s, h)	17.34±3.18	18.08±3.67	1.445	0.150
FBG (x̄±s, mmol/L)	8.59±3.13	4.99±1.36	11.908	<0.01
TG n (%)				
≥7.0 mmol/L	42(60.86)	38(25.17)	26.089	<0.01
<7.0 mmol/L	27(39.14)	113(74.83)		
LDL-C (x̄±s, mmol/L)	2.45±1.07	2.60±0.97	1.030	0.304
HDL-C (x̄±s, mmol/L)	1.12±0.33	1.13±0.29	0.227	0.821
Hcy (x̄±s, μmol/L)	13.59±3.43	9.37±3.41	8.501	<0.01
CRP (x̄±s, mg/L)	8.25±2.97	4.26±2.39	10.623	<0.01
Hb (x̄±s, g/L)	134.87±19.13	137.01±17.74	0.810	0.419
RBC (x̄±s, ×10^12^/L)	4.55±0.69	4.47±0.60	0.875	0.383
PLT (x̄±s, ×10^9^/L)	208.06±70.77	212.35±72.36	0.411	0.682
NE (x̄±s, %)	0.65±0.10	0.64±0.11	0.643	0.521
LY (x̄±s, %)	0.25±0.09	0.27±0.13	1.157	0.249
NIHSS on admission (x̄±s, min)	8.46±2.82	3.62±2.63	9.224	<0.01
ASPECT (x̄±s, points)	6.50±0.85	8.64±0.81	17.901	<0.01

A logistic regression model was created to analyze the risk factors for early neurological deterioration in IS patients, with END as the dependent variableand single-factor P<0.05 as the independent variable.The findings indicated that the history of diabetes (OR=1.398, P=0.301), TG (OR=6.149,P<0.05), ASPECT score (OR=7.641, P<0.05), FBG (OR=2.172, P<0.05), CRP (OR=1.706, P<0.05), NIHSS (OR=1.336, P<0.05), and Hcy (OR=1.425, P<0.05) were independent risk factors for early neurologicaldeterioration in IS patients (P<0.05).Details are presented in Table 2.

**Table 2:** Logistic multivariate analysis of early neurological deterioration in IS patients.

Influencing factors	β	SE	Wald χ^2^<br>value	*P*	OR	95%CI	
						Lower limit	Upper limit
History of diabetes	0.335	0.324	1.068	0.301	1.398	0.741	2.637
TG≥7.0 mmol/L	1.816	0.259	49.342	0.000	6.149	3.704	10.207
ASPECT	2.076	0.284	55.621	0.000	7.641	4.643	13.664
FBG	0.776	0.111	48.642	0.000	2.172	1.746	2.701
CRP	0.534	0.075	50.130	0.000	1.706	1.472	1.978
NIHSS	0.352	0.054	46.063	0.000	1.336	1.258	1.569
Hcy	0.354	0.055	41.998	0.000	1.425	1.280	1.587

**Figure 1:**
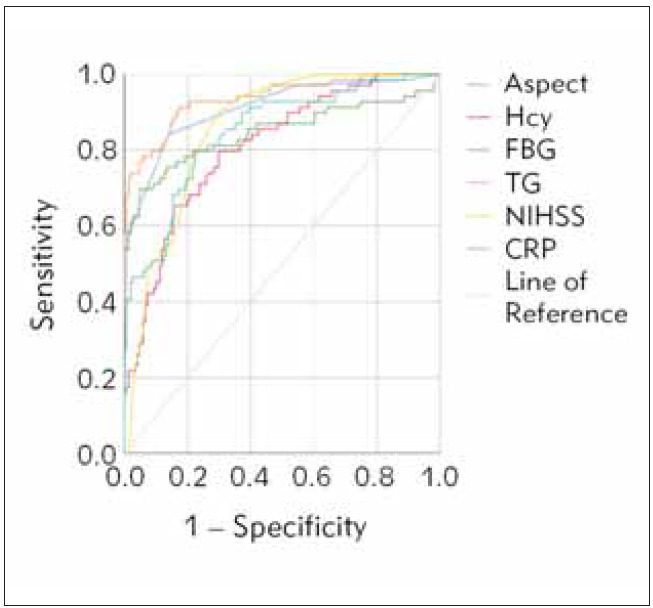
END risk ROC curve analysis. Note: The closer the ROC curve shape is to the upper left corner (0, 1), the better the model’s performance. The AUC value ranges from 0 to 1, with values closer to 1 indicating better model performance.

ROC analysis yielded an ASPECT area under the curve of 0.910 (95% CI:0.864 to 0.944), sensitivityof 84.06%, specificity of 86.09%, Hcy of 0.808 (95% CI:0.750 to 0.858), sensitivity of 79.71%, specificity of 70.20%, and FBG of 0.847 (95% CI:0.793 to 0.892). The sensitivity was 69.57%, specificity 95.36%, TG 0.937 (95% CI: 0.896–0.965), sensitivity 91.30%, specificity 82.78%, NIHSS 0.857 (95% CI: 0.803–0.900), sensitivity 89.86%, and specificity 70.20%, indicating that the calibration curve suggests that the model has good consistency. See Table 3 and Figure 1 for details.

**Table 3:** ROC curve analysis of ASPECT, Hcy, FBG, TG, and NIHSS. Note: AUC is the area under the ROC curve.

Risk factors	AUC	*P* value	95%CI		Youden index	Sensitivity	Specificity
			Lower limit	Upper limit			
ASPECT	0.910	<0.001	0.864	0.944	0.702	84.06	86.09
Hcy	0.808	<0.001	0.750	0.858	0.499	79.71	70.20
FBG	0.847	0.000	0.793	0.892	0.649	69.57	95.36
TG	0.937	0.000	0.896	0.965	0.741	91.30	82.78
NIHSS	0.857	0.000	0.803	0.900	0.601	89.86	70.20

### Establishment of the nomogram prediction model

**Figure 2:**
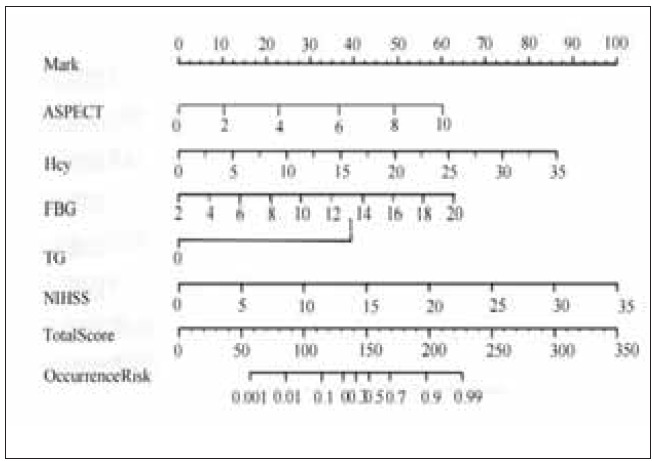
Nomogram of IS post-ENDrisk prediction model. Note: The vertical line represents each variable, while the horizontal line indicates the total score or predicted risk value. The score line is determined according to the weight and importance of each variable, which is distributed in a stepped manner at the top of the nomogram. Each variable’s contribution to the total score can be understood by its position on the scoreline. The scores for the individual variables are added together to obtain a total score. The total score line is a horizontal line at the bottom of the nomogram, connecting the score lines and clearly showing the relative importance of each variable in the overall score. The predicted risk value is obtained on the prediction line at the bottom of the nomogram based on the total score. These forecast lines are usually created using historical data or professional expertise to help understand the predicted risk of the target variable at different total scores.

A nomogram model predicting the risk of IS post-END was developed based on the logistic multivariate regression analysis results. Each of the 9 predictive factors was given a preliminary score, with a total score ranging from 0 to 100 points. These were then summed to predict the risk of END in patients (0–100%) based on the total scores. The constructed nomogram model showed that ASPECT was 59.2, Hcy was 84.0, FBG was 61.4, TG (≥7.0 mmol/L) was 39.4, and NIHSS was 98.1, with a total score of 345.7. Details are presented in Figure 2.

**Figure 3:**
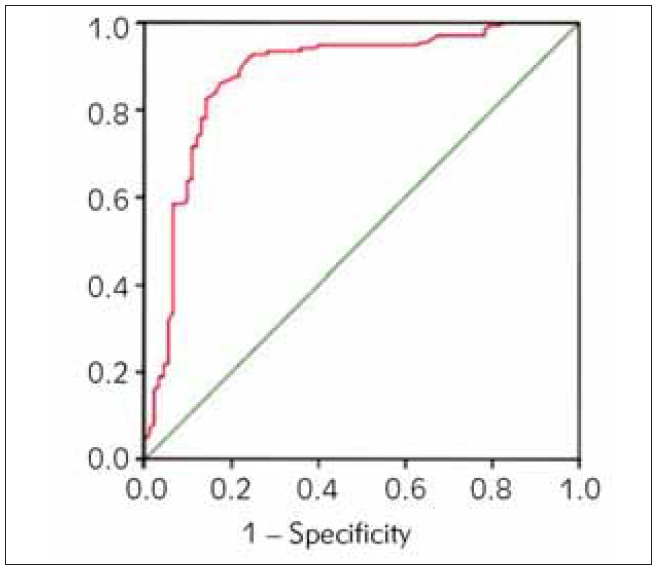
ROC curve assessment nomogram model.

The Hosmer-Lemeshow goodness of fit test was applied to evaluate the model. The results showed χ^2^=0.509, P=0.812, indicating a good fit and strong interpretative ability. The ROC curve assessedthe model’s discrimination, showing an area under the curve of 0.879 (95% CI: 0.824–0.942), a sensitivity of 86.9%, and a specificity of 83.5%, suggesting high accuracy in distinguishing between diseased and non-diseased individuals. Repeated sampling with the Bootstrap method yielded an area under the partial calibration curve of 0.849 (95% CI: 0.791–0.903), a sensitivity of 85.4%, and a specificity of 80.1%, demonstrating the model’s stability. The C-index of the prediction model is 0.872, 0.863, and 0.854 in internal validation, Bootstrap method, and external validation, respectively, indicating that the model has strong generalization ability. Details are presented in Figure 3.

## Discussion

END is one of the key markers of early disease progression in IS patients, and its extremely high disability and mortality rates seriously affect the prognosis of IS patients, making the prevention and treatment of END a major challenge for the treatment of IS [17]. Although some progress has been made in understanding the pathogenesis and risk factors of END in the medical community, it remains unclear, and there is a lack of accurate and reliable prediction methods, posing significant challenges to the prevention and treatment of END in IS patients. Therefore, conducting an in-depth analysis of the risk factors for END in IS patients is crucial, as well as predicting and assessing the risk of END early and guiding early prevention and control accordingly [18]. This approach helps delay disease progression and provides a strong guarantee for improving the prognosis of IS patients, which has significant clinical importance. Understanding the risk factors of END in IS patients and systematically identifying and analyzing them is essential for early detection of high-risk patients, developing relevant interventions, and reducing the incidence of END. Secondly, establishing and improving the prediction model of END in IS patients makes it possible to assess patients’ risk more accurately. This model can quantitatively assess END risk by combining various information such as patients’ clinical symptoms, imaging examination results, and laboratory test data. It is helpful for clinicians to formulate treatment plans more specifically and improve the prevention and treatment effect. According to the risk assessment results of END, high-risk patients need active drug therapy, such as antiplatelet drugs and anticoagulants; lifestyle interventions, such as smoking cessation and alcohol restriction, reasonable diet, increased exercise; and necessary rehabilitation treatment to delay early interventions, such as neurological deterioration [19].

Our study identified diabetes, TG, ASPECT score, FBG, CRP, NIHSS, and Hcy as independentpredictors of END in AIS patients, Whereas a study by Sung et al. found that the initial NIHSS score, hemorrhagic transformation, and stenosis or occlusion were significant predictors of END following stroke thrombectomy [19]. As well as our study, Sabir Rashid et al. [20] study found that hyperglycemia is a predictor of END. In this paper, by univariate and logistic multivariate analysis, it was found that the previous history of diabetes and TG were independent risk factors for END in patients, mainly because long-term diabetes would lead to decreased arterial elasticity, diffuse changes in cerebrovascular and reduced blood perfusion. This increases the possibility of brain ischemia and hypoxia, which may lead to neurological dysfunction more easily. Hyperglycemia causes significant hemodynamic changes in patients with diabetes, leading to abnormal plasma proteins and elevated fibrinogen, in addition to the failure of blood flow autoregulation, further exacerbating the risk of neurological dysfunction. Long-term diabetes also leads to atherosclerosis, which thickens the microvascular basement membrane and prevents the microvascular endothelium from working properly [20]. This condition usually occurs in the large, medium, and small arteries, so infarcts are mostly located deep in white matter with less collateral circulation, thus increasing the risk of neurological dysfunction. In diabetic patients, the formed components of the blood will change, leading to many aggregated red blood cells, reduced fluidity, decreased deformability, and increased adhesion. This results in a hypercoagulable state, which raises the risk of neurological dysfunction [21]. Elevated TG levels may lead to increased blood viscosity and slower blood flow, which affects the blood supply to the brain. Abnormal TG levels may cause damage to vascular endothelial cells, triggering inflammatory reactions and atherosclerosis, further exacerbating brain ischemia and neurological dysfunction. It may affect cellular signalling and gene expression, causing damage to the nervous system [21]. Abnormal TG levels may increase oxidative stress in the blood, leading to neuronal damage and dysfunction.

The results indicated a positive correlation between ASPECT and NIHSS with early END (P<0.05). This is so logical for scoring systems designed for risk stratification in AIS patients. ASPECT score reflects the degree of brain ischemia in IS patients. Higher scores indicate less ischemia and less likelihood of neurological impairment. Con - versely, the lower the score, the more severe the ischemia and the greater the likelihood of neurological impairment. The ASPECT score was closely related to the lesion size. The larger the lesion, the lower the ASPECT score, which may lead to more severe neurological dysfunction [22]. The severity of vascular stenosis significantly impacts the ASPECT score. Severe stenosis can result in insufficient cerebral blood supply, leading to brain tissue damage and impaired neurological function. Hypertension and hyperglycemia are known risk factors for IS. Poor management of blood pressure and glucose levels can worsen cerebral ischemia, lower ASPECT scores, and elevate the risk of neurological dysfunction. During the acute phase, stroke patients may experience rapid deterioration. If timely or adequate treatment is not provided, it may lead to a reduction in ASPECT scores and an increase in neurological impairment [23]. The impact of NIHSS on early neurological dysfunction in IS patients is significant. The NIHSS score reflects the severity of cerebral ischemia; higher scores indicate more severe ischemia, which can result in neurological dysfunction. Additionally, higher NIHSS scores predict worse patient prognosis, potentially increasing the risk of neurological dysfunction. The NIHSS score can also predict factors related to early neurological dysfunction, such as age, hypertension, and diabetes. Therefore, early NIHSS assessment in IS patients helps predict the risk of early neurological dysfunction and guides appropriate treatment measures to reduce its incidence (Matsuzono et al., 2021).

Through logistic regression analysis, we found that FBG and CRP were the main influencing factors for early END (P<0.05). There is a certain dependence of brain tissue on glucose utilization. After IS, energy metabolism disorders in brain tissue lead to decreased glucose utilization and impaired nerve cell function, leading to neurological dysfunction.

Multivariate analysis also found that higher Hcy levels increased END probability (P<0.05). Hcy can damage the vascular endothelium and coagulation system, resulting in ischemic cerebrovascular disease. It has neurotoxic effects and may directly cause cognitive impairment after stroke [24]. Homocysteine (Hcy) promotes the oxidation of low-density lipoprotein cholesterol, encourages the formation of foam cells, thickens blood vessel walls, and narrows vessel width, which can easily lead to thrombosis [24].

The Bootstrap repeated sampling method was employed to split the dataset into training and validation sets. A model was constructed using the training set and evaluated with the validation set. The evaluation metrics included sensitivity, specificity, accuracy, and positive and negative predictive values. Internal validation demonstrated that the nomogram model possesses high predictive accuracy and substantial clinical application value. Further analysis revealed that ASPECTS is a scoring system designed to assess early CT findings in stroke patients, with scores ranging from 0 to 10; lower scores indicate more severe patient conditions. In this study, the patient’s ASPECT score was 59.2. Homocysteine (Hcy) is an amino acid closely associated with the development of cardiovascular and cerebrovascular diseases, and elevated Hcy levels are considered a risk factor for END. The patient’s Hcy score in this study was 84.0. Fasting blood glucose (FBG) levels are crucial for evaluating diabetic patients. In this study, they are also linked to END, with the patient’s FBG score being 61.4. However, certain subgroups of patients with special clinical features may require further correction or the development of specific risk prediction models.

## Conclusion

The nomogram model of IS post-END risk prediction constructed in this study has been verified to have high prediction accuracy through rigorous experimental design and data analysis. This result is of great practical value to clinicians and provides strong decision support. In practical clinical work, doctors can identify high-risk patients early using this model to perform targeted intervention and treatment. Early identification and targeted intervention for high-risk patients are anticipated to reduce the incidence of END post-IS. END can severely impact the quality of life and can be life-threatening; thus, reducing its incidence improves patients’ prognosis and quality of life, which is undoubtedly a significant benefit for patients and their families. However, it is important to note that this was a single-centre study, and the results were obtained from specific populations and regions. This means that in the future, multi-centre validation is necessary to evaluate this model’s applicability and promotion value in different areas and populations. Therefore, further studies are needed to explore other risk factors affecting IS post-END.

## Dodatak

### Fundings

The research is supported by the Clinical Scientific Research Fund project of Zhejiang Provincial Medical Association (No. 2022ZYC-A93) and the Science and Technology research project of Jinhua City, Zhejiang Province (No. 2022-4-264).

### Conflict of interest statement

All the authors declare that they have no conflict of interest in this work.

### List of abbreviations

END, early neurological deterioration; 

FBG, fasting blood glucose; 

TG, triglycerides; 

LDL-C, lowdensity lipoprotein cholesterol; 

HDL-C, high-density lipoprotein cholesterol; 

Hcy, homocysteine; 

CRP, C-reactive protein; 

Hb, haemoglobin; 

RBC, red blood cell count; 

PLT, platelet count; 

NE, neutrophil ratio; 

LY, the lymphocyte ratio; 

NIHSS, the National Institutes of Health Stroke Scale in the United States; 

ASPECT, CT score; 

IS, ischemic stroke.
